# ENRICHING THE LIVES OF OLDER VETERANS: THE GEROFIT WAY

**DOI:** 10.1093/geroni/igac059.1629

**Published:** 2022-12-20

**Authors:** Katherine Hall, Adam Gepner

## Abstract

Gerofit is a clinical exercise program for Veterans ages 65 years and older with multi-morbidities and functional limitations that place them at elevated risk for institutionalization. It was declared a VHA “Best Practice” in 2017 and was selected for widespread dissemination and implementation, with over 30 Gerofit programs spanning the country. The original Gerofit program consists of supervised, facility-based exercise, offered 3 days per week in a group setting. Each exercise prescription incorporates aerobic training, progressive resistance training, and specialized exercises for balance and physical function. Everything changed in March 2020 when facilities began receiving orders to shut down face to face encounters and social distancing measures were put in place. Within six weeks, all of our sites had transitioned to group-based virtual classes, aptly called Gerofit to Home (GTH). This symposium includes 2 presentations that explore the effect of GTH on physical functioning and health-related quality of life outcomes in veteran participants during the pandemic: one focusing on resilience among GTH adopters vs. non-adopters in patients formerly participating in facility-based Gerofit; and the second focusing on patients enrolled directly in to GTH (no facility-based experience). The third presentation describes differences in physical function and health gains among Gerofit participants with and without peripheral artery disease (PAD). The fourth and fifth presentations describe augmented models of care to enrich the Gerofit experience for a) participants with supplemental nutrition programming and b) advanced physical therapy trainees through a Gerofit resident training model.

